# Vitamin D Status and Its Consequences for Health in South Africa

**DOI:** 10.3390/ijerph13101019

**Published:** 2016-10-18

**Authors:** Mary Norval, Anna K. Coussens, Robert J. Wilkinson, Liza Bornman, Robyn M. Lucas, Caradee Y. Wright

**Affiliations:** 1 Biomedical Science, University of Edinburgh Medical School, Edinburgh EH8 9AG, UK; Mary.Norval@ed.ac.uk; 2 Clinical Infectious Disease Research Initiative, Institute of Infectious Diseases and Molecular Medicine, University of Cape Town, Cape Town 7701, South Africa; anna.coussens@uct.ac.za (A.K.C.); robert.wilkinson@uct.ac.za (R.J.W.); 3 Department of Medicine, Imperial College London, London SW7 2AZ, UK; 4 Mill Hill Laboratory, Francis Crick Institute, London NW1 2BE, UK; 5 Department of Biochemistry, Faculty of Science, University of Johannesburg, Gauteng 2006, South Africa; lizab@uj.ac.za; 6 National Centre for Epidemiology and Population Health, The Australian National University, Canberra, ACT 2601, Australia; Robyn.Lucas@anu.edu.au; 7 South African Medical Research Council, Environment and Health Research Unit and University of Pretoria, Department of Geography, Geoinformatics and Meteorology, Pretoria 0001, South Africa

**Keywords:** 25(OH)D levels, HIV-1, tuberculosis, vitamin D receptor, sun exposure

## Abstract

In this review, reports were retrieved in which vitamin D status, as assessed by serum 25-hydroxyvitamin D [25(OH)D] levels, was measured in South African population groups with varied skin colours and ethnicities. Healthy children and adults were generally vitamin D-sufficient [25(OH)D level >50 nmol/L] but the majority of those aged above 65 years were deficient. A major role for exposure to solar ultraviolet radiation (UVR) in determining 25(OH)D levels was apparent, with the dietary contribution being minor. Limited data exist regarding the impact of recent changes in lifestyles on vitamin D status, such as urbanisation. With regard to disease susceptibility, 11 of 22 relevant publications indicated association between low 25(OH)D levels and disease, with deficiency most notably found in individuals with tuberculosis and HIV-1. Information on the relationship between vitamin D receptor variants and ethnicity, disease or treatment response in the South African population groups demonstrated complex interactions between genetics, epigenetics and the environment. Whether vitamin D plays an important role in protection against the range of diseases that currently constitute a large burden on the health services in South Africa requires further investigation. Only then can accurate advice be given about personal sun exposure or dietary vitamin D supplementation.

## 1. Introduction

Vitamin D deficiency is a risk factor for rickets in children and osteomalacia in adults [1]. Recently, with the recognition that the active form of vitamin D is immunoregulatory, vitamin D deficiency has been proposed to increase the likelihood of a wide range of common human disorders [2,3], including various autoimmune, infectious and cardiovascular diseases, and internal cancers, several of which occur in South Africa at rates exceeding those reported globally. While there has been considerable research on vitamin D in developed countries [4,5], this has not been matched in developing countries where the impact of vitamin D deficiency on health may be greater in terms of disease burden. Therefore in this review, we gather together all the published information regarding vitamin D in the context of South Africa. Our main aims were to establish whether there was widespread vitamin D deficiency in any population group and to investigate whether there was robust evidence to associate low vitamin D status with any of the diseases, prevalent currently in South Africa. 

In most people, exposure of the skin to solar ultraviolet radiation (UVR) in the UV-B range (290–315 nm) is the main route for synthesis of vitamin D. Absorption of UVR converts 7-dehydrocholesterol in the lower epidermis to previtamin D_3_ which then undergoes thermal isomerisation to vitamin D_3_. This is followed by hydroxylation, first to 25-hydroxyvitamin D [25(OH)D] mainly in the liver, and then to 1,25-dihydroxyvitamin D [1,25(OH)_2_D] mainly in the kidney [6]. Both 25(OH)D and 1,25(OH)_2_D are transported in the blood linked to the vitamin D binding protein (DBP, also known as group-specific component [Gc]). 1,25(OH)_2_D binds to the vitamin D receptor (VDR) in the membrane and/or the nucleus of a wide variety of cell types. In addition many non-renal cells can convert 25(OH)D to 1,25(OH)_2_D, thus providing a source of 1,25(OH)_2_D for tissue-specific intracellular action [7]. Binding of 1,25(OH)_2_D facilitates the VDR to form a heterodimer with the retinoid X receptor (RXR), leading to the activation of the VDR-RXR transcription complex. This regulates the expression of more than 900 genes, thus affecting a myriad of signalling cascades in the body [8].

Vitamin D is present in some foods, as vitamin D_3_ (cholecalciferol) from animal sources and as vitamins D_3_ and D_2_ (ergocalciferol) from plant sources. Both forms are absorbed from the small intestine together with dietary fat, and incorporated into chylomicrons which are rapidly transported via the lymphatics and then via the venous blood, bound to the DBP, to the liver. A limited number of staple foodstuffs, such as cereals and dairy products, are fortified with vitamin D in many developed countries. Finally dietary supplements are widely available, containing either vitamin D_2_ or D_3_ in a wide range of dosages.

Vitamin D status is commonly measured as the total plasma or serum concentration of the sum of 25(OH)D_2_ and 25(OH)D_3_, both bound to DBP and albumin, as well as unbound (“free”) [9]. The cut-off points for sufficiency, insufficiency and deficiency are not agreed at present. In their 2011 report, the US Institute of Medicine focussed on bone health and defined <30 nmol/L 25(OH)D as deficient, 30–50 nmol/L as insufficient and >50 nmol/L as sufficient [10]. Others such as Bischoff-Ferrari et al. [11] and the US Endocrine Society [12] have defined <50 nmol/L as deficient, 50–75 nmol/L as insufficient and >75 nmol/L as sufficient, based on the skeletal and non-skeletal health benefits of vitamin D. In the following section, a 25(OH)D concentration of >50 nmol/L in people without disease was taken as being sufficient as the levels relating to bone are not generally disputed, while those relating to the other possible in vivo benefits of vitamin D remain uncertain. It should be noted that the accuracy and reproducibility of the assays that measure 25(OH)D are variable [13,14]. At present quality assurance-validated liquid chromatography-tandem mass spectroscopy (LC-MS/MS) is considered by many as the “gold standard” method [13], but the lower-cost competitive immunoassay, which varies by supplier, is often used and may give different results from LC-MS/MS, thus confounding comparison between studies.

**Figure 1:**
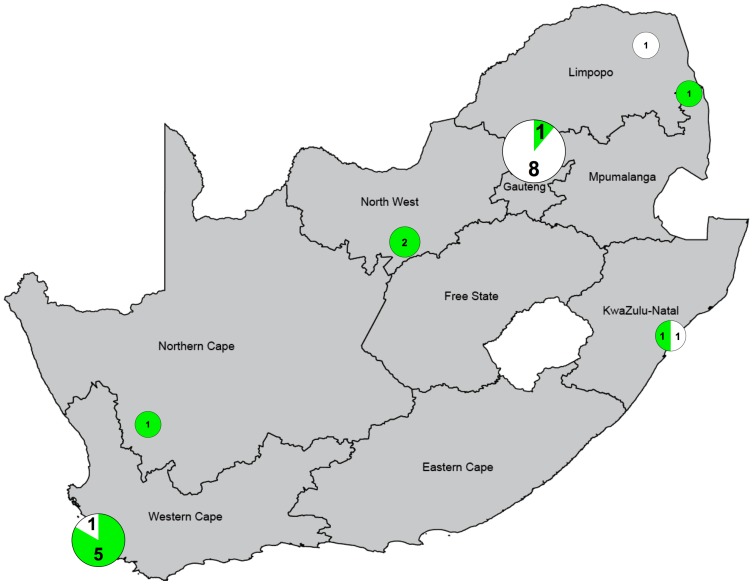
The number of publications investigating the association between vitamin D status and disease in the Provinces of South Africa. Green indicates a significant association, and white indicates no significant association. The size of the Venn diagrams is proportional to the number of studies. Map adapted from Global Security [19] and the Venn diagrams were plotted using GraphPad PRISM 6.

South Africa consists of nine provinces, spanning latitudes 22.34° S to 34.28° S (Figure 1). The country has a narrow coastal plain at sea level, rising to mountains over 3000 m in height, with a large plateau, the Highveld, at an altitude of 1200 m. High atmospheric pressure over this Northern plateau frequently results in cloudless skies and, in combination with the altitude, leads to high levels of ambient solar UVR. As an illustration of the climatic variation between the Northern and Southern regions of South Africa, the temperature, UV Index, and number of hours of sunshine per day in the winter and summer are shown in Table 1 for Cape Town representing the South, and Pretoria representing the North Highveld. These conditions have the potential to result in variable seasonal personal exposure to solar UVR, with implications for the production of vitamin D [15].

The population of South Africa is multi-ethnic and complex, with phototypes ranging from deeply pigmented to fair [20]. The South African government collects demographic data on four ethnic groups: in 2014, the National Census estimated the population as 54 million of whom 80.2% were Black African, 8.8% Coloured (mixed ancestry between White and Black or Black and Asian, with skin colour ranging from pale to dark brown), 2.5% Asian/Indian (frequently termed Indian in South Africa) and 8.4% White [21]. Approximately 10% of the population is infected with HIV-1 currently, one of the highest prevalences in the world, and the incidence of tuberculosis (TB) is the third highest in the world. Epidemiological transition in South Africa, occurring particularly since the end of apartheid in 1994, means that many social factors relating to personal sun exposure and hence to vitamin D status may have changed in recent years. 

In this article, published studies measuring the vitamin D status of various healthy South African populations are reviewed first. These provide information to indicate whether any population group, defined by age or ethnicity or location of residence, is vitamin D-deficient. Such a status may lead to deleterious consequences for health. It is important to try to construct simple messages for the public regarding “safe” sun exposure that ensures sufficient vitamin D status while not increasing the risk of the adverse effects of intense UVR. This topic is considered in the context of the large range of South African phototypes. Next, as urbanisation may affect vitamin D status, either positively through improvements in lifestyle and diet, or negatively through less time spent outdoors, this aspect is reviewed. Information regarding disease and vitamin D deficiency is then evaluated in the context of South Africa. This country has a high burden of diseases such as TB and HIV, so the possibility of a low vitamin D status increasing the risk of the disorder or its severity is of particular interest. Finally recent information is reviewed regarding the effect of the *VDR* polymorphisms, found in the diverse populations of South Africa, on susceptibility to disease, particularly TB. 

## 2. Vitamin D Status in South Africa

Several recent reviews have provided information on global vitamin D status [4,5,22], but all of these lack data for most African countries, including South Africa. In this section, all the publications were retrieved in which 25(OH)D was assessed with the aim of determining whether any South African population group, defined by age or skin colour or place of residence, was vitamin D-deficient. A systematic search was conducted using PubMed, covering the period 1978 to mid-2015, with the terms “vitamin D” AND “South Africa” AND “(status or prevalence or deficiency)”. By title, 220 papers were found; this number was reduced to 85 after reading the abstracts, and further reduced to 19 after considering the full reports: these are listed in Table 2 with a summary of each set of findings. Publications involving case reports, clinical management and reviews were excluded. Reports assessing 25(OH)D levels in people with various diseases and the control groups are described in a later section.

The results were compared for variation in the methods assessing 25(OH)D level, sample size, the age and population groups of the participants, the latitude of the study location and the time of year of sampling. Several different assays were used to determine the concentration of 25(OH)D in plasma or serum, ranging from competitive protein binding to LC-MS/MS more recently. Results from these different methods may not be comparable. Sample sizes tended to be smaller in older reports compared with more recent studies. The findings of each investigation varied, and included people at only one location. The sampling frames are indicated in Table 2, and show that they were limited to particular groups and did not reflect the demographics of the population as a whole. Thus none of the results in the 19 publications could be considered as providing an accurate reflection of the entire South African population. The participants varied in age from infants to the elderly, and were stated as belonging to the Black African, Coloured, Asian/Indian or White population groups in all but three cases [31,39,40]. Residence ranged from latitude 24–35° S, within both rural and urban communities, but it should be noted that only five of the studies were based in the South of the country. Samples were collected throughout the year in seven investigations and, of these, 25(OH)D levels were assessed in each of the four seasons in five [25,28,31,36,39], with a mean value for the year given in the other two [33,37]. The season of sampling was stated as being solely in the winter in three instances [23,39,41] and at the end of the summer in another one [26]. There was one longitudinal study in which 25(OH)D levels were assessed in the summer and the following winter in the same participants [38]. It is particularly important to obtain this information because 25(OH)D levels may decrease in the winter months, especially in the southern part of South Africa, as the quantity of UV-B in solar UVR reduces. In the remaining eight investigations, the time of year when the samples were collected was not specified.

Despite these difficulties, an overview of the studies in Table 2 indicates that 25(OH)D levels were generally sufficient in most healthy children (birth to 18 years old) with black, coloured and white skin (mean value approximately 84 nmol/L) [23,24,26,28], but were about 24% lower in Black African children compared with age-matched White children [28]. It is of note that all of these studies were located in the Northern provinces of South Africa and that four of them were conduced prior to 1986 [27,28,29,30] so their relevance to the current vitamin D status of children throughout South Africa who are undergoing rapid urbanisation, and particularly to those living in the Southern provinces is uncertain. In addition the results using assays for 25(OH)D at that time may not correlate with the assays used currently. The levels of 25(OH)D were sufficient in most healthy Black African (mean approximately 67 nmol/L) and White (mean approximately 65 nmol/L) adults (aged 18–54 years) [29,30,32,37], but insufficient in many Asian/Indians (mean approximately 42 nmol/L) [33,35,36], hypothesised to be due to the cultural preference to clothe most of the body. Three studies undertaken prior to 1997 in elderly people (>65 years) showed a high prevalence of vitamin D deficiency (mean approximately 38 nmol/L) [39,40,41]. However in the only study since then which included data on the elderly, a mean level of 65 nmol/L in healthy Black African women over the age of 70 years was found [32]. Males tended to have higher 25(OH)D levels than age-matched females in two recent reports [28,33], and the levels decreased with age, both during adolescence [27], and in adults [32]. Levels were higher in the summer/autumn (peak in autumn) compared with the winter/spring [33,36,39], and, in the only longitudinal study, decreased from sufficiency in the summer to less than 50 nmol/L in the winter in both the Black African and Coloured groups in Cape Town [38]. They also tended to decrease with increasing distance from the Equator [37].

## 3. Sun Exposure and Photoprotection in Relation to Vitamin D Production in South Africa

The Cancer Association of South Africa produced a Vitamin D Sun Exposure Statement in 2014 [42]. While this document details dietary sources of vitamin D and mentions links between vitamin D deficiency and certain diseases, it does not provide guidance on how much time to spend in the sun to produce sufficient vitamin D in South Africa, and does not distinguish between people with fair and darkly pigmented skin. In fact, there is no scientifically validated and agreed safe threshold level of sun exposure that allows for optimal vitamin D synthesis without increasing the risk of harm. Chronic unprotected personal exposure to solar UVR and excess exposure leading to sunburn are associated with several adverse health effects, including skin cancer and cataract [43]. Personal photoprotection, such as broad-brimmed hats, clothing, shade and sunscreen, is recommended by the World Health Organization during peak solar UVR hours around midday, and at any time when the UV Index is ≥3 [44]. A combination of these methods reduces over-exposure effectively. Studies carried out predominantly in people with fair skin living in Australia, USA and Europe indicate that sunscreen usage does not affect 25(OH)D levels significantly [45], although a small decrease occurs if they are applied at frequent intervals and at the correct thickness, neither of which are common practice [46].

A systematic search using PubMed with the terms “sun exposure/sun protection” AND “South Africa” AND “vitamin D” identified 18 articles by title. After excluding case and clinical management reports and reviews, and consideration of the full papers, the number was reduced to nine. One study in Durban (latitude 30° S) showed that children, aged 6 years, received about 5% of the total daily ambient solar UVR [47], similar to that received by children in Australia [48] and England [49]. A nationwide study among primary schoolchildren in South Africa suggested that about half of the children ever wore a hat and/or used sunscreen [50]. Even amongst those at high risk of sunburn and skin cancer, as is the case for individuals with oculocutaneous albinism, personal photoprotection is frequently minimal [51,52].

As already indicated, several reports based in South Africa demonstrate a major role of solar UVR in determining vitamin D status [28,33,36,38,39]. Daily sunshine hours by season were noted in a study of the vitamin D status of children in Johannesburg but were not analysed in relation to 25(OH)D level [28]. One in vitro study showed that exposure of vials containing 7-dehydrocholesterol to sunlight for one hour at different times on one day in each month of the year resulted in the same quantity of vitamin D being produced in Johannesburg throughout the year, but the quantity was reduced to about one-third during the winter months in Cape Town compared with the summer months at this location [15]. It was suggested that the decrease in the vitamin D status of people living in Johannesburg in the winter months may be due to the drop in temperature, and consequently increased clothing and decreased time spent outdoors, rather than to the reduction in ambient solar UVR. Two studies, one in Johannesburg and the other in Cape Town, have quantified the participants’ recollection of daily sun exposure during the past week using a questionnaire, and then related this to their 25(OH)D level [36,38]. Daily sun exposure scores, based on duration of time spent outdoors and amount of skin exposed, were positive predictors of 25(OH)D concentration among Black Africans in Johannesburg, and season and sun exposure explained 17% of the variance in 25(OH)D concentration [36]. Daily sun exposure scores did not predict 25(OH)D concentration among Asian/Indian participants who were mainly veiled females. In the Cape Town populations, personal sun exposure was the strongest determinant of 25(OH)D level, driven by the area of skin exposed and duration of exposure [38]. Black African participants spent about 4 h longer each week in the sun in both winter and summer than Coloured participants, and they exposed larger areas of their bodies in the summer, while both groups reduced their body exposure to similar levels in the winter [38]. 

## 4. Effect of Urbanisation and Diet on Vitamin D Status in South Africa

African countries are amongst the fastest urbanising in the world [53]. In South Africa, official government projections estimate that, between 2011 and 2016, the provinces of Gauteng and Western Cape will experience an inflow of 1.5 million migrants, many from the Eastern Cape and Limpopo [21]. Generally these people feel driven to move because of population growth and associated economic and social pressures. The situation has worsened over the past 25 years due to large-scale intensive farming at the cost of traditional small-scale subsistence-based production. With urbanisation, changes in lifestyle occur which may lead to reduced personal solar UVR exposure, with the potential to decrease vitamin D status. For example, office work is less conducive to time spent in the sun, and urban environments, such as tall buildings and narrow streets, may limit direct sun exposure. As urbanization frequently leads to changes in the diet which may, in turn, affect vitamin D status, these two aspects are considered in this section. 

A systematic search was conducted using PubMed for papers published in English, with no limit on years and with the terms “vitamin D” AND “South Africa” AND “rural” OR “urbanisation” OR “semi-rural”. By title 165 articles were found. After excluding duplicates and studies that were not about South Africa or vitamin D, 50 papers remained which were reduced to 15 after reading the abstracts. These provided relevant information on the diet of South African populations, defined as living in urban, semi-rural or rural settings. Papers were excluded if they focused on vitamin D in relation to a disease outcome, if they did not present original data, or if they did not specifically present results for vitamin D intakes or serum 25(OH)D levels. 

Only two investigations compared 25(OH)D levels in rural and urban populations in South Africa. In a study of Black African women in the North West Province in 2011, Kruger et al. [32] found that serum 25(OH)D levels were lower in urban women aged between 50 and 70 years (urban: 63 nmol/L; rural: 71 and 75 nmol/L, depending on age decade), but similar between urban- and rural-living women <50 years and >70 years. More than 30 years earlier, Pettifor et al. [24] analysed 25(OH)D levels in 60 black children living in rural, small urban and large urban communities near Johannesburg. The mean values were 72.3, 77.3 and 82.8 nmol/L respectively, thus indicating a small increase in vitamin D status with urbanisation. However it should be noted that the sample size was small, the assay may have given different results from the assays currently employed, the standard deviations were large and, as the children were of primary school age, they might spend considerable time outdoors, even in an urban environment.

Dietary intake of vitamin D is low in African countries in both urban and rural environments, and the contribution of 25(OH)D_2_ to the overall 25(OH)D status is minor [36,37]. In South Africa, many margarines are fortified with vitamin D (one brand contains 9200 IU/Kg) but other foods are not, and few foods available to the majority of the population contain significant amounts of vitamin D. In rural KwaZulu-Natal, the mean dietary vitamin D intake was considerably less than the dietary recommended intake of 15 μg/day (600 IU/day) [54,55]. Although the diets of urban Black African women in the north-west Provinces of South Africa were healthier than those of rural women, all of the participants had low vitamin D intakes (means from 1.79 to 3.28 μg/day), and urban women were more likely to be obese [32]. Elderly Black African people in a peri-urban environment near Cape Town also had low average vitamin D intake [56], as did healthy Black African and Coloured 18–24 year olds in the same region [38]. Low vitamin D intake was consistently found in both Black African (median 2.96 µg/day) and Asian-Indian (median 1.17 µg/day) adults in the Johannesburg area, with a marked difference in the prevalence of vitamin D supplementation use: 1.9% of Black African adults and 14.9% of Asian-Indian adults [36]. 

Low intake of vitamin D and calcium have been hypothesised as a cause of the high prevalence of stunting in children aged 2–5 years (36.9% had stunted growth) in a semi-urban impoverished setting in the Hantam district of the Northern Cape Province [57]. Similar low vitamin D intake was reported amongst 5-year old children living in the urban Johannesburg/Soweto area [58].

With such limited information, it is not possible currently to determine whether urbanisation with or without associated changes in the diet leads to an overall increase or decrease in the vitamin D status of an individual in South Africa. In addition the definition of what constitutes urban and rural may differ between the studies described above.

## 5. Association of Vitamin D Status with Diseases in South Africa

One of the major research interests in vitamin D at the present time lies in investigating the possible association of low 25(OH)D levels with various diseases. This aspect is considered in detail below in the context of South Africa, and is of particular relevance to the infectious diseases commonly found in this country. A systematic search was conducted using PubMed, covering 1973 to mid-2015, with the terms “vitamin D” AND “South Africa” AND “(disease or health or infection)”. By title, 117 papers were found; this was reduced to 25 after reading the abstracts, and to 22 after reading the full reports. Papers were excluded for six reasons: a review or commentary; non-South African population; no in vivo data; no disease association; not human; 25(OH)D not measured. Details of each study are shown in Table 3 and their geographic location in Figure 1. It is notable that 15 of these papers have been published since 2011, reflecting the recent rise of interest in vitamin D and health outcomes. The most common diseases studied were bone pathologies and infectious disease (ID), each representing 9 of the 22 reports, with one investigating bone growth and vitamin D status in HIV-1-infected women [62]. The threshold for vitamin D sufficiency and deficiency as defined in each of these articles was used as it was not possible to re-calculate statistical significance using one standard value from the data presented. 

Of the 22 studies, 11 found a significant inverse association between 25(OH)D level and disease presentation, severity or pathogenesis (Table 3). It should be noted that, in four of the other 11 studies that did not find association, the comparison was between HIV-1-associated disease states, such as HIV-1 vs. HIV-1-cryptococcal meningitis [63], rather than between disease vs. healthy control, and vitamin D deficiency was present in 42%–74% of the HIV-1 infected persons. The significant inverse correlation between disease presentation and 25(OH)D levels was more prevalent in studies conducted in southern and western provinces where, due to the higher latitude in the south and lower altitude in the south and west compared with the central/northern Highveld, there is less solar UV-B radiation in the winter months, and thus the likelihood of seasonal deficiency is greater (Figure 1). The association between disease and seasonal sunlight hours was highlighted in three studies, two indicating lower incidences of TB and TB meningitis following the summer increase in solar UVB radiation [64,65]. The third showed a decrease in HIV-1 replication in peripheral blood mononuclear cells (PBMCs), collected in the summer and infected ex vivo in autologous serum, compared with HIV-1 replication in PBMCs from the same individual collected in the winter [38].

Aside from the accepted link between vitamin D deficiency and bone mineralisation, the recent focus of vitamin D research in South Africa on HIV-1 and TB reflects the burden of these chronic diseases in this country. Seasonal vitamin D deficiency was significantly associated with disease or HIV-1 replication in three studies from the Western Cape [38,64,65], with vitamin D deficiency more severe in patients co-infected with HIV-1 and *Mycobacterium tuberculosis* [64]. Three multinational cohort studies which included participants from Gauteng and/or KwaZulu-Natal also found vitamin D deficiency in approximately 40%–50% of HIV-1-infected patients, but there was no control comparison group so that disease association could not be assessed [67,68,69]. Participants from the PEARLS (Prospective Evaluation of Antiretrovirals in Resource Limited Settings) trial were used in two of these studies. At 24 weeks after initiation of antiretroviral therapy (ART), patients receiving efavirenz-containing regimes had significantly decreased serum 25(OH)D concentration, with the greatest decrease occurring in those with higher baseline 25(OH)D levels. Notably ART did not improve micronutrient deficiency in HIV-1-infected persons [67,68].

Vitamin D deficiency may be the result of both HIV-1 infection and loss of HIV-1 killing mechanisms with a further decrease in CD4+ T cells [72]. The HIV-1 viral envelope protein gp120 induces CYP27B1 expression [73], the enzyme which metabolises 25(OH)D, indicating that infection may contribute to decreased circulating 25(OH)D and vitamin D deficiency. There is also a variety of immune pathways regulated by vitamin D, leading to inhibition of HIV replication [74,75,76]. As well as this antimicrobial activity, vitamin D can also reduce HIV replication by affecting the production of factors, such as NF-kB, which are required for transcription of the long terminal repeat of HIV-1 [77,78,79]. 

The nine studies on bone health included six investigating rickets, stunting or metabolic bone disease in children and adolescents in the Northern and Eastern Provinces [22,25,57,59,61]. Three found significant association between low vitamin D intake and disease, or clinical improvement following three months of vitamin D supplementation (5000 IU/day) [25,57,59]. In the three that did not detect association, there was a high prevalence of vitamin D deficiency in the children investigated, particularly in those hospitalised. It is possible that the duration of deficiency could be an important determinant of disease development [25].

Bone mineral density (BMD) and vitamin D status were also examined in two studies of older adults in Gauteng and North-West Province [32,35]. No association between 25(OH)D levels and BMD was demonstrated in either report. However the concentration of parathyroid hormone (PTH), which upregulates 1,25(OH)_2_D production from 25(OH)D, thus increasing intestinal calcium absorption, was inversely correlated with BMD in Asian-Indian participants in Gauteng [35]. In Black African women in the North-West Province, 25(OH)D levels were inversely correlated with the concentrations of PTH and C-terminal telopeptide of Type 1 colleagen (CTX, a measure of bone resorption) in rural participants; urban participants had lower CTX levels which correlated with dietary calcium and PTH, the PTH in turn correlating with 25(OH)D. This suggests that urban women might be at risk of high bone resorption in response to calcium insufficiency and low vitamin D status [32].

Three of the 22 reports considered association between cardiovascular disease or obesity/metabolic syndrome and 25(OH)D levels. The incidences of cerebrovascular disease, heart disease, and type 2 diabetes mellitus (T2DM), representing the third, fourth and fifth highest cause of mortality in South Africa, after TB and influenza/pneumonia, are all increasing currently, indicating their importance to the health of the country [80]. Only one study investigated vitamin D status and indicators of cardiovascular disease, finding that women with low 25(OH)D levels had significantly higher systolic blood pressure [34]. Using the same cohort of adults in greater Johannesburg, elevated PTH, not low 25(OH)D, predicted metabolic syndrome, although 25(OH)D inversely correlated with PTH [33,34]. It should be noted that it is not possible to separate cause and effect from such cross-sectional studies, and there many be confounding by factors such as physical activity which may relate to both time in the sun and disease risk. Any link between vitamin D status and T2DM in South African populations has not been investigated thus far. In addition, although cancer causes 8.3% of all deaths in South Africa, no association with vitamin D status has been sought. This is despite some evidence supporting a role for vitamin D in the regulation of cell differentiation, angiogenesis and matrix remodelling, all key components of metastasis [81].

Other non-communicable outcomes investigated included the management of zinc deficiency in Alzheimer’s [59], and the association between alcohol use disorders and vitamin D status in adolescents [60]. The former study found that adjunct vitamin D supplementation together with zinc and vitamin A was more effective at increasing zinc levels, than zinc alone [70], suggesting an interaction between micronutrients. Chronic alcohol use in Western Cape adolescent 12–16 year olds was significantly associated with lower serum 25(OH)D and calcium levels and all the participants had inadequate dietary intakes of vitamin D [71]. 

## 6. Vitamin D Receptor Gene (*VDR*) Polymorphisms and Ethnicity in South Africa

The *VDR*, the gene encoding the vitamin D receptor protein (VDR), spans more than 100 kb on chromosome 12. Besides eight protein-coding exons *(2–9)*, almost half of the *VDR* sequence serves regulation, including six, largely noncoding, exons *(1a–1f)*. Four promoters and four enhancers regulate transcription from the *VDR* and alternative splicing delivers at least nine splice variants encoding different proteins. Having nine *bona fide* CpG islands (CGIs) and three miRNA recognition elements in its 3’ UTR, *VDR* is regulated in a complex fashion through genetics, epigenetics and environment [82]. *VDR* polymorphisms most commonly studied include *Fok*I, *Bsm*I, *Apa*I and *Taq*I, named after restriction enzyme cleaving sites. Their alleles are referred to as either letters or nucleotides, such as *Fok*I (rs2228570, previously rs10735810, C/T or F/f [lower case indicating restriction site presence]), *Bsm*I (rs1544410, A/G or A/a), and *Taq*I (rs731236, T/C or T/t).

Different ethnicities in South Africa have evolved at different geographical locations under diverse conditions. Selection factors established unique combinations of genetic and epigenetic variants for optimal function of genes and pathways, including that of vitamin D [83,84]. Recent lifestyles changes which lead to less exposure to solar UVR, together with DBP and *VDR* genetic variants with low affinity for 25(OH)D, the frequency of which differs between population groups, may contribute to disease susceptibility. 

In the following section, information on the *VDR* variants found in the populations of South Africa was collated, with the aim of determining the function of such polymorphisms in relation to the risk of disease or an association with ethnicity. Reports on the association of *VDR* variants with ethnicity, disease or treatment response, *VDR* DNA methylation and VDR function in South Africans were identified using the PubMed search terms: VDR polymorphism AND South Africa AND (disease OR health OR infection OR infectious OR seasonal OR ethnicity OR pigmentation) OR (seasonal variation AND infectious disease AND polymorphism AND pigmentation AND nutrition). Of the eleven papers identified, seven were relevant experimental reports investigating *VDR* polymorphisms in South African populations (Table 4). Five related to disease [38,86,87,89,90], and two to ethnicity alone [85,88]. The first study on *VDR* single nucleotide polymorphisms (SNPs) in South African Whites, Blacks and Asian-Indians showed a significantly higher frequency of *Taq*I “TT” and ApaI “AA” genotypes in Blacks compared to Whites and Asian-Indians [85]. A case-control study in the Black Venda people living in Limpopo Province found no independent *VDR* SNP association with pulmonary TB, but protection by *Fok*I-*Bsm*I-*Apa*I-*Taq*I haplotype, “F-b-A-T”, proposed to encode a VDR variant with more effective transactivation of target genes [86]. In a South African Coloured population in the Western Cape, *VDR* polymorphisms were associated with time to conversion of sputum to smear negativity during chemotherapy of patients with pulmonary TB, but there was no association with susceptibility to TB [87]. A second study of the Venda revealed a complex association of *Taq*I with the methylation status of the 3’ region CGI 1060 of the *VDR*, in which site-specific methylation distinguished ethnicity and TB status [88]. In South African Black children, the minor “T” (f) allele of *Fok*I correlated with susceptibility to respiratory syncytial virus infection [89], as similarly reported in Dutch children [91]. A recent study of Black African and Coloured adults in Cape Town found *Fok*I “AG” associated with lower serum 25(OH)D levels, as well as a lower 25(OH)D status in winter and following supplementation with vitamin D [38]. The impact of *Fok*I on VDR function was examined using monocytes from healthy South African Blacks and Whites [90]. Despite having higher levels of more active VDR, monocytes from Blacks showed reduced transactivation of the gene encoding the cathelicidin antimicrobial protein (CAMP), compared to Whites, unless supplemented in vitro with 1,25(OH)_2_D_3_. Thus the association of *VDR* polymorphisms with disease in the population groups of South Africa appears to be ethnicity-dependent, involving complex interactions between genetics, epigenetics and the environment. 

## 7. Conclusions

A systematic search of the published literature provided limited but important information relating to the vitamin D status of the South African population. The study protocols were varied, involving males and females of different ages, ethnicities, skin colours and places of residence, together with a range of methods to measure 25(OH)D levels. Despite these considerable shortcomings, 25(OH)D levels were generally found to be sufficient (>50 nmol/L) in most healthy people, although they were frequently insufficient in Asian/Indians and in the elderly (>65 years) and it should be noted that little data are available for those living at the higher southern latitudes of the country, especially during the winter months. The few studies investigating whether urbanisation might lead to an increase or decrease in vitamin D status have yielded contradictory results. A combination of factors is involved here: for example, less exposure to the sun might be predicted in urban compared with rural environments with a consequent reduction in 25(OH)D levels, but on the other hand, urbanisation might lead to a higher standard of living with the consumption of more foods containing vitamin D and its metabolites. A low dietary intake of vitamin D occurs throughout South Africa, emphasising the importance of exposure to the sun in determining vitamin D status. The use of vitamin D supplements amongst South Africans is not common.

Reports investigating vitamin D status in relation to disease, have yielded inconsistent results, with half finding a relationship between 25(OH)D deficiency and disease or its severity, and half finding no such association. The majority of the studies falling into the latter category were in the North of the country where the mean 25(OH)D levels tended to be higher compared with the South (Figure 1, Table 3). Most studies were cross-sectional or case-control so that establishing the temporal relationships and thus revealing whether low 25(OH)D levels are a cause, consequence or proxy for a “true” risk factor is not possible. Similarly, the sample sizes tended to be small, especially in the earlier reports, and were underpowered to detect an effect on disease risk. Furthermore the population groups and ages varied, and the disorders covered a very wide range, with bone pathologies and IDs being most common. It is of interest that 1,25(OH)_2_D interacts with cells of the immune system including monocytes/macrophages, dendritic cells, B and T cells, leading to modulation of many inflammatory responses through various signalling pathways [91]. Inflammation and infection can associate with low 25(OH)D levels, and linkage studies may identify consequence rather than cause. Whether vitamin D deficiency is causal to disease or not, the significant burden of deficiency in individuals with a variety of disorders in South Africa suggests that vitamin D supplements should be part of the therapy, at least to maintain bone health but also possibly to enhance recovery and disease resolution. In addition it is becoming clear that not all the immunomodulatory effects of exposure to solar UVR are due to vitamin D [92]. Further work in this area is merited, particularly concentrating on the disorders that present major health burdens in South Africa currently, such as HIV, TB, cardiovascular diseases, T2DM and various cancers. 

It is not possible at the present time to give accurate advice to the general public, based on scientific findings, regarding how much sun exposure is optimal for health. There are many environmental variables to take into consideration, such as the latitude of residence, season, time of day, cloud cover and surface reflectivity. In addition variations in personal factors, including skin pigmentation and the extent of clothing, require that the health messages are adapted to make them relevant for each ethnic group. Given the climate of South Africa, there should be ample opportunity to attain vitamin D sufficiency throughout the year for those living in the northern Provinces and in all but the winter months for those living in the southern Provinces when there is limited UVB in sunlight. Our review shows that some populations are at increased risk of vitamin D deficiency. These include the elderly, those in residential care, Asian-Indian women who tend to be fully clothed, those with deeply pigmented skin, and people infected with HIV and *M. tuberculosis*. It is possible that the obese and workers employed underground or on night shifts may also be at risk of deficiency. All, or at least some, of these groups may benefit from vitamin D supplementation.

## Figures and Tables

**Table 1:** Weather conditions in Cape Town and Pretoria; data from [16,17,18].

Parameter	Cape Town *Latitude 35° S, Altitude 0–300 m*		Pretoria *Latitude 26° S, Altitude 1339 m*	
	Summer	Winter	Summer	Winter
Average hours of sunshine per day	11	5	10	8
Ultraviolet index	9–10	2–3	11^+^	4–6
Average temperature in °C, minimum/maximum	16/26	7/18	18/28	5/20

**Table 2:** Studies assessing vitamin D status, as 25-hydroxyvitamin D [25(OH)D] levels, in various populations of children, adults and the elderly in South Africa (listed chronologically by year in each section).

Study Population, Sampling Frame	Age (Years)	Location (Latitude), Season of Sampling when Specified	Assay	Serum or Plasma 25(OH)D Concentration Means (Unless Specified), nmol/L	Reference (Year)
*Children*					
285 Coloured (mixed race) children; community based	1–17	Western Township, Johannesburg (26° S), winter	Competitive protein binding	78.0 in age 1–12 y, 58.5 in age 13–14 y, 56.8 in age 15–16 y; none <25; no difference between boys and girls	[23] (1978)
60 Black African children; school sample	7–12	Rural, small urban and large urban communities near Johannesburg (26° S)	Competitive protein binding	72.3 in rural, 77.3 in small urban, 82.8 in large urban; none <25	[24] (1979)
114 hospitalised Black African infants; random hospital admissions	0–2	Witwatersrand (26° S), throughout year	Competitive protein binding	37.3 aged 1–24 months, no correlation with age or season; <12.5 in 7%. 19.8 aged 0–1 month, probably reflecting vitamin D status of mother	[25] (1985)
20 Black African pre-school children; cluster sample of villages	3–5	Villages in Northern Transvaal, now Limpopo (24° S), end of summer	Competitive protein binding	85.5, no difference between underweight and normal weight children	[26] (1986)
82 Black Africans with OCA, 58 Black Africans; school sample	6–18	Pietersburg, Northern Province (24° S)	Radioimmunoassay	125 in OCA Blacks, 103 in Blacks, 6–9 y; 116 in OCA Blacks, 86.3 in Blacks, 10–13 y; 90.3 in OCA Blacks, 90.8 in Blacks,14–18 y	[27] (2000)
295 Black African children, 90 White children; bone health subcohort of Birth-to-Twenty longitudinal cohort	10	Johannesburg (26° S), all seasons	Chemiluminescence (DiaSorin Liaison)	100 in Black boys, 129 in White boys, 86 in Black girls, 112 in White girls; <50 in 8% Blacks and 1% Whites; higher values in White children in summer/autumn than in winter/spring, no seasonal variation in Blacks	[28] (2011)
*Adults*					
43 healthy Black African women and cord blood of their babies, shortly after delivery; hospital based	16–40	Transkei (31° S)	Competitive protein binding	81.8 in mothers; 171 in cord blood	[29] (1987)
105 healthy White and 74 Black African premenopausal nurses; 50 healthy White and 65 Black African postmenopausal nurses; hospital based	20–64	Witwatersrand (26° S)	Competitive protein binding	Medians–65.8 in premenopausal White, 48.3 Black; 64.5 in postmenopausal White, 67.5 Black	[30] (1997)
216 requests for vitamin D testing (39% with suspected osteoporosis); hospital based	All ages, peaks at 2 and 64	Western Cape (32° S), all seasons	Competitive protein binding	Medians–48.3 (range 5.5–106); <45 in 41%; no seasonal effect on level	[31] (2009)
658 rural healthy Black African women, 603 urban healthy Black African women; random selection from Prospective Urban and Rural Epidemiology Study or community based	>35	North West Province (27° S), rural and urban seasonally matched	Roche Eledsys 2010 COBAS system	Levels decreased with age in both rural and urban women from about 78 at <50 y to about 65 at >70 y; levels lower in urban than in rural women, aged 50–70 y	[32] (2011)
373 Black Africans, 344 Asian/Indians; cohort from Birth-to-Twenty longitudinal study	Mean 42	Johannesburg-Soweto (26° S), all seasons	HPLC	70.9 Blacks, 41.8 Asian/Indian; <30 3% Blacks and 13% Asian/Indians; females lower than males in both groups; highest level in autumn	[33] (2013)
291 healthy urban Black African women; random selection from Prospective Urban and Rural Epidemiology Study or community based	>47 (mean 57.6)	North West Province (27° S)	Roche Elecsys 2010 COBAS system	65; those with levels <75 two-times more likely to have higher systolic blood pressure than those with >75 (151 vs. 146 mmHg)	[34] (2013)
368 healthy Black Africans and 347 healthy Asian/Indians; random selection from Birth-to-Twenty longitudinal study	18–65	Johannesburg (26° S)	HPLC	58.3 in Black females, 72.7 in Black males, 35.7 in Asian/Indian females, 45.4 in Asian/Indian males	[35] (2014)
371 healthy Black Africans and 343 healthy Asian/Indians; random selection from Birth-to-Twenty longitudinal study	18–65	Johannesburg (26° S)	HPLC	56.8 in Black females, 72.4 in Black males, 32.4 in Asian/Indian females, 43.9 in Asian/Indian males; <30 in 5% Blacks and 28.6% Asian/Indians; levels 40%–60% higher in autumn than in winter/spring; little 25(OH)D_2_	[36] (2014)
502 Black Africans; population sample from Modelling of the Epidemiologic Transition Study	25–45 (mean 33.4)	Cape Town (34° S), winter and summer months	LC-MS/MS	59.3; <30 in 6.6%, >50 in 65.9%; negative correlation of 25(OH)D level with distance from the equator (by comparing levels in Blacks living at latitudes 41° N, 17° N, 6° N, 4° S and Cape Town)	[37] (2014)
50 healthy Black Africans, 50 healthy Coloured (Cape mixed); community based longitudinal study	18–24	Cape Town (34° S), winter and summer months	Chemiluminescence (DiaSorin, Liaison)	Medians: 72.6 Black, 65.5 Coloured in summer; 45.5 Black, 43.8 Coloured in winter	[38] (2015)
*Elderly*					
232 patients with femoral neck fractures, ethnicity not specified; hospital admissions	Mean 72.7	Johannesburg (26° S), throughout year	Competitive protein binding	44.3 throughout year; 51 in summer/autumn, 38.1 in winter and spring; <25 in 17% subjects in winter/spring	[39] (1978)
60 females living in old-age homes, ethnicity not specified	Mean 80	Pretoria (26° S), winter	Not specified	32	[40] (1991)
173 non-institutionalised Coloured (mixed race), 52% women; population sample	65–92 (mean 73.7)	Cape Town (34° S), late winter	Not specified	37; <25 in 17%	[41] (1996)

HPLC, high-performance liquid chromatography; LC-MS/MS, liquid chromatography-tandem mass spectroscopy; OCA, oculocutaneous albinism; y, years.

**Table 3:** Vitamin D deficiency and disease associations in South African populations, by disease, ethnicity, location, age and gender.

Category	Disease/Study Groups	Assoc ^1^	Study Type	Location	Population	Sample Size	Age Group	Gender	Reference
Bone	Fractures	Yes	longitudinal	Gauteng	NA	20, 20	6–29 y	M & F	[59]
Bone	Rickets	Yes	cross-sectional	KwaZulu-Natal	Black African	37	1–12 y	M & F	[60]
Bone	Bone mineral density	Yes	cross-sectional	North-West	Black African	658	>45 y	F	[32]
Bone	Growth stunting	Yes	cross-sectional	Northern Cape	NA	150	2–5 y	M & F	[57]
Bone	Rickets	No	cross-sectional	Gauteng	Black African	114	<2 y	M & F	[25]
Bone	Metabolic bone disease	No	cross-sectional	Gauteng	Black African	26	16–19 y	M	[61]
Bone	Bone mineral density	No	cross-sectional	Gauteng	Black & Asian-Indian	371, 343	18–65 y	M & F	[35]
Bone	Under weight vs. normal weight	No	case-control	Limpopo	Black African	145	3–5 y	M & F	[22]
Bone/ID	BMD in HIV uninfected vs. HIV high CD4 vs. HIV low CD4 count	No	case-control	Gauteng	Black African	98, 74, 75	≥18 y	F	[62]
ID	Schistosomiasis (PZQ vs. PZQ + vitamin D vs. vitamin D vs. placebo)	Yes ^2^	RCT	Mozambique border	NA	14, 16, 14, 15	14–18 y	M	[63]
ID	TB-Meningitis	Yes ^3^	case-control	Western Cape	Black & Coloured	42, 147	0–13 y	M & F	[64]
ID	HIV replication (Summer vs. winter vs. winter + vitamin D)	Yes	longitudinal	Western Cape	Black African	30	18–24 y	M & F	[38]
ID	TB HIV (TB vs. HIV vs. TB-HIV vs. OD)	Yes	case-control	Western Cape	Black African	93, 75, 99, 103	≥18 y	M & F	[65]
ID	HIV-Cryptococcal Meningitis vs. HIV	No ^4^	case-control	Western Cape	NA	150, 150	≥21 y	M & F	[66]
ID	HIV ART initiation	NA ^5^	cross-sectional	Gauteng/KWN	NA	270	≥18 y	M & F	[67]
ID	HIV ART initiation	NA ^5^	cross-sectional	Gauteng/KWN	NA	270	≥18 y	M & F	[68]
ID	Paradoxical TB-HIV IRIS vs TB-HIV no IRIS	No	case-control	KwaZulu-Natal	Black African	11, 11	24–50 y	M & F	[69]
ID	ART-associated TB vs HIV+TB-	No	case-control	KwaZulu-Natal	Black African	18, 38	23–57 y	M & F	[69]
NCD	Cardiovascular disease (blood pressure and pulse)	Yes	cross-sectional	North-West	Black African	291	>47 y	F	[34]
NCD	Metabolic syndrome	No	cross-sectional	Gauteng	Black & Asian-Indian	374, 350	18–65 y	M & F	[33]
NCD	Obesity (total body fat, fat distribution)	No	cross-sectional	Gauteng	Black & Asian-Indian	371, 343	18–65 y	M & F	[36]
Nutrition	Alzheimer’s zinc deficiency (Zn vs. Zn ± vitamin A ± vitamin D)	Yes ^6^	RCT	Western Cape	NA	70/group	55 y	M	[70]
Nutrition	Alcohol use disorders vs. matched controls	Yes	cross-sectional	Western Cape	Mixed ancestry	81, 81	12–16 y	M & F	[71]

Assoc., association; ART, antiretroviral therapy; BMD, bone mineral density; C:C, case vs. control; CVD, cardiovascular disease; F, female; HIV, human immunodeficiency virus; ID, infectious disease; IRIS, immune reconstitution inflammatory disease; M, male; NA, not available; NCD, Non communicable disease; OD, other diseases; PZQ, praziquantel; RCT, randomised controlled trial; TB, tuberculosis. Bold indicates significant association. y, year; ^1^ Disease prevalence or disease-associated functional response negatively correlated with serum 25(OH)D levels or positively with vitamin D deficiency. ^2^ Vitamin D supplementation improved lymphocyte and eosinophil function in schistosomiasis-infected individuals, disease association not investigated. ^3^ Winter sunlight hours associated with TB-meningitis. ^4^ 74% deficiency in HIV-meningitis patients, but no difference to HIV-only. ^5^ Multinational cohort (*n* = 30 of 270 from South Africa).HIV disease association not assessed (40%–50% deficient); initiation of Efavirenz containing ART regimes significantly decreased 25(OH)D levels. ^6^ Improved plasma zinc levels in combination Zn + vitamin A + vitamin D.

**Table 4:** Vitamin D receptor gene (*VDR*) variants in South Africans, associated with ethnicity, disease, treatment response, DNA methylation, *VDR* expression, vitamin D receptor protein (VDR) level or VDR transactivation of target genes.

SNP (rs)	Study Population; Gender when Specified	Factor Investigated	Study Design	Median/Mean Age, Years (Range)	Association	Reference
*Fok*I (rs2544037) (rs10783219) (rs10735810) *Taq*I (rs731236)	22 male, 28 female Cape mixed; 26 male, 24 female Black Africans (Zhosa)	Determinants of 25(OH)D status, before and after vitamin D supplementation	Longitudinal	(18–24)	*Fok*I (rs10735810) “AG” contributed to lower 25(OH)D, and lower 25(OH)D in winter and post-supplementation, while “AA” contributed to higher 25(OH)D, in a linear model incorporating UVB exposure, skin pigmentation, ethnicity, age and sex	[38]
*Apa*I (rs7975232) *Taq*I (rs731236)	264 Black Africans, 247 Whites, 194 Asian-Indians	Ethnicity	Retrospective cross-sectional cohort of healthy male and female blood donors	NA	Higher *Apa*I “AA” and *Taq*I “TT” in Blacks than Whites or Asian-Indians; no difference between Whites and Asian-Indians. Higher frequency of *Taq*I “T” may contribute to lower incidence of osteoporosis in Blacks	[85]
*Fok*I (rs2228570) *Bsm*I (rs1544410) *Apa*I (rs7975232) *Taq*I (rs731236)	95 Black Africans (Venda) with TB, 117 ethnicity matched controls	TB	Case-control	NA	No independent SNP association; *Fok*I-*Bsm*I-*Apa*I-*Taq*I haplotype “F-b-A-T” (C-G-T-T) protects	[86]
*Fok*I (rs2228570) *Apa*I (rs7975232) *Taq*I (rs731236)	All Colored: 249 TB cases, 352 ethnicity matched controls, 220 TB cases converting from positive to negative	TB and chemotherapy for TB	Case-control for TB; longitudinal for TB conversion	(18–65)	No association of *VDR* allele, genotype, haplotype or diplotype with TB. Quicker response to TB chemotherapy in *Apa*I “AA” (TT) vs. “aa” (GG), in *Taq*I “Tt” (TC) vs. “tt” (CC) and in *Taq*I “TT” (TT) vs. “tt” (CC)	[87]
*Apa*I (rs7975232) *Taq*I (rs731236)	15 male, 15 female CAU (North America); 15 male, 15 female YRI (Nigeria); 16 male, 16 female Black Africans (Venda) with TB together with 12 male and 17 female healthy Black African controls	Ethnicity, TB, and DNA methylation of the *VDR* 3’ region CGI 1060	Ethnicity; case-control for TB	CAU 32 (22–44), YRI unknown, TB cases 38 (18–62) TB controls 34 (21–62)	Methylation variable positions in 3’ end of *VDR* distinguish ethnicity and TB status. Higher regional methylation in *Taq*I “TC/CC” than “TT” in YRI and Venda, but not in CAU	[88]
*Fok*I (rs10735810 merged into rs2228570)	296 Black Africans with RSV, 113 Black African controls	RSV-related disease	Case-control	Cases 3.0 months, controls 3.5 months	*Fok*I “C” frequency higher in Black South Africans than European, Asian and Japanese populations. “CT” and “T” predispose to RSV disease, while “CC” protects	[89]
*Fok*I (rs2228570)	40 healthy Black Africans, 20 healthy Whites	*VDR* expression and VDR function, considering ethnicity and 25(OH)D levels	Cross-sectional cohort of male and female blood donors	35 (17–65)	Higher frequency *Fok*I “CC” genotype and VDR protein in Blacks than Whites, but less *VDR* mRNA and *CAMP* mRNA. *CAMP* mRNA higher in *Fok*I “CT/TT” genotypes than “CC”. Circulating 25(OH)D levels ≥50 nmol/L, and comparable between Blacks and Whites. Different effects on *CAMP* and *CYP24A1* expression in Whites and Blacks on supplementation with 1,25(OH)_2_D	[90]

1,25(OH)_2_D: 1,25-Dihydroxyvitamin D; 25(OH)D: 25-Hydroxyvitamin D; *CAMP*: Gene coding cathelicidin antimicrobial protein; CAU: Caucasian; CGI: CpG island; *CYP24A1*: Gene coding cytochrome P450 family 24 subfamily A member 1; NA: Not available; RSV: Respiratory syncytial virus; SNP (rs): Single nucleotide polymorphism (reference SNP cluster ID to access a specific SNP in the dbSNP database); TB: Tuberculosis; UVB: Ultraviolet B radiation; YRI: Yoruba (African ancestry, Ibadan, Nigeria).
